# Unleashing excellence: using a project management approach to effectively implement a simulation curriculum to improve residents’ preparedness

**DOI:** 10.1186/s12909-024-05166-y

**Published:** 2024-03-04

**Authors:** Claudia Ebm, Carolina del Pozo, Andrea Barbarello, Giovani Poli, Stefania Brusa

**Affiliations:** 1 Department of Biomedical Sciences, Humanitas University, Pieve Emanuele, Milan Italy; 2 IRCCS Humanitas Research Hospital, Rozzano, Milan Italy

**Keywords:** Implementation science, Simulation curriculum, Education, Postgraduate training, Quality improvement, Patient safety, Emergency preparedness

## Abstract

**Background:**

Integrating innovative, simulation-based training programs into medical curricula frequently encounters familiar challenges, including scepticism, limited faculty time, and financial constraints. Recognized for its success in business projects, the Harvard Project Management Theory emerges as a promising approach to optimizing the implementation process and achieving sustainable success. This study endeavours to elucidate the application of project management theory in our implementation process and assess its impact on the clinical preparedness of novice residents.

**Methods:**

The research utilized a structured four-phase implementation strategy—Planning, Build-up, Execution, and Closing—to develop a simulation-based education curriculum. Incorporating project management tools like project charters and risk management tools played a crucial role in facilitating the effective implementation of standardized processes and improved clinical outcomes. Essential components of this innovative management approach encompass stakeholder engagement, milestone definition, and the alignment of institutional policies and processes.

**Results:**

A collective of 395 residents actively engaged in eight monthly simulation-based events, reflecting an average participation rate of 39 residents per lecture (± 19). A noteworthy enhancement was observed in the average rating for knowledge gain, with a significant improvement from 5.9/10 to 8.8/10 (*p* = 0.0001). Participants highlighted the program’s considerable impact on future clinical practice (4.7/5) and teamwork (4.8/5) as particularly valuable aspects. The introduction of a novel organizational structure received favourable feedback from faculty members, with a notable rating of 4.8/5 for predictive time planning. Qualitative insights from the evaluation highlighted the significance of targeted incentive schemes in optimizing the implementation process.

**Conclusion:**

This project underscores the constructive influence of project management principles in designing simulation-based curricula, explicitly focusing on stakeholder engagement, faculty motivation, and data utilization. Adopting the Harvard Project Management Approach emerges as a catalyst for heightened success in curriculum design, contributing to enhanced emergency preparedness among novice residents. The positive outcomes observed in this study provide valuable insights for future implementations, offering a foundation for refining and optimizing medical education programs to meet the evolving needs of learners and stakeholders alike.

**Supplementary Information:**

The online version contains supplementary material available at 10.1186/s12909-024-05166-y.

## Background

Simulation-based training has emerged as a highly effective approach for improving the clinical preparedness of medical residents, particularly in emergency and critical care settings [1–4]. By participating in simulated clinical scenarios, residents can apply their theoretical knowledge, develop critical thinking skills, and make clinical decisions while receiving immediate feedback and debriefing [2]. This approach also allows residents to reflect on their performance, identify areas for improvement, and gain a deeper understanding of patient care. As technological advancements continue to drive the evolution of simulations, these technologies’ enhanced realism and immersive nature offer residents a more authentic clinical experience, ultimately leading to improved learning outcomes and better patient care [5–7]. Several studies have confirmed the effectiveness of simulation-based training in improving new residents’ clinical skills, knowledge, and confidence [8–12].

Despite these well-described benefits, the successful implementation of simulation-based projects faces several challenges. Typical barriers include the high costs associated with acquiring and maintaining simulation equipment and software, significant time commitments from faculty and learners, resistance to changing the educational delivery mode, lack of training or experience with simulation technology, and challenges integrating simulation training into existing curricula [12–16]. These barriers can make running and sustaining a simulation training program difficult, but they can be overcome with proper planning, human resource management, investments, and real-time testing. Under such conditions, implementation science has emerged successfully [17, 18] as a systematic approach to improve efficiency, leading to greater acceptability, sustainability, and scalability. It provides guidance, theories, tools, and strategies for implementing programs effectively, measuring outcomes, and considering the implementation context. In particular, implementation science aims to bridge the gap between theory and practice, enables the translation of evidence into practical applications, and improves health and educational outcomes while maximizing research investments [19].

We hypothesized that using a structured approach to achieve targeted educational outcomes may increase the success and sustainability of our curriculum. The Harvard Project Management Theory, originally designed to support business projects, holds the potential for managing and implementing such complex projects and hence may lead to improved preparedness of medical residents facing emergencies [20, 21].

This paper describes how we used Harvard Project Management Theory to design a new innovative curriculum introduced at Humanitas University in 2021. As such, this paper seeks to evaluate the effectiveness of the Harvard Project Management Approach in enhancing curriculum design success and ultimately positively impacting residents’ emergency preparedness.

## Methodology

In 2021, we launched SIMCLUB, a cross-disciplinary curriculum centred around simulation-based learning. The idea was tailored for residents across twelve Humanitas University and IRCCS Humanitas Research Hospital residency schools. The project’s conception was prompted by the increasing hospital demand for residents to manage emergency scenarios with confidence and efficiency.

### Project management intervention

**Fig. 1:**
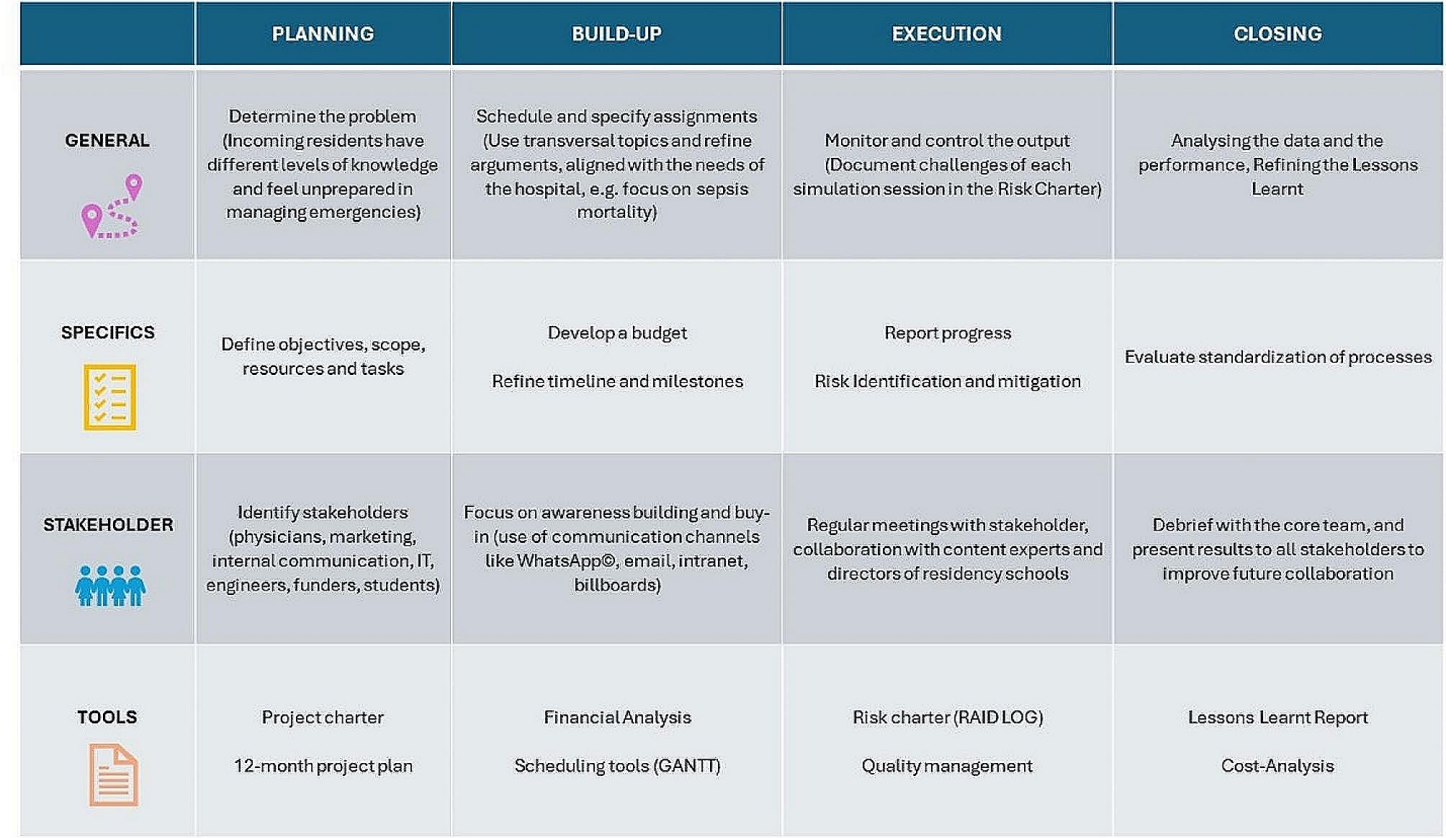
This figure illustrates the specific actions, stakeholders, and tools used to structure the distinct phases of the project effectively. *RAID: Risks, assumptions, issues, and dependencies *GANTT chart: illustrates work completed over a period about the time planned for the work

In order to orchestrate the project effectively, we chose a methodical approach, implementing the Harvard Project Management Theory for comprehensive structuring, tracking, and delineation of its evolution. Adhering to this methodology, we segmented the simulation-based training curriculum into four phases: Planning, Build-up, Execution, and Closing. Complementary to this framework, we developed essential tools, including a project charter, project plan, risk management plan, and project monitoring and control mechanisms. The delineation of these phases, specific steps, and identification of critical stakeholders pivotal for attaining success are elucidated in Fig. 1.

### Project phases

#### Phase 1: Planning phase

At the outset of a project, the initiation phase serves as the foundation. This stage involves defining the project’s purpose, scope, and objectives. The primary emphasis is on acquiring the initial insights necessary to comprehensively understand the project’s goals. Notably, a needs assessment is conducted to identify clinical gaps and areas for improvement in the medical setting. Identifying key stakeholders and assessing their expectations, requirements, and potential risks are imperative during this phase. The culmination of this stage involves the creation of a project charter, setting the trajectory for the project, and authorizing its commencement.

##### Recommended tools

SWOT Analysis, Stakeholder Analysis, Feasibility Studies.

#### Phase 2: Build-up

The planning phase comes into play once the project’s parameters are set. Here, the core team dives deeper into the project’s details, develops the simulation topics, and refines, together with clinicians and design consultants, the clinical objectives and assessment methods. Schedules of the simulation courses are established, resources are allocated, and training courses are initiated. It is about creating a roadmap that guides the team from start to finish. Risk management strategies are developed, and communication plans are implemented to ensure everyone is aligned with the project’s objectives. Incentive schemes are designed, tested, and implemented. The output of this phase is a comprehensive plan that serves as a blueprint for the entire project.

##### Recommended tools

Gantt Charts.

#### Phase 3: Execution phase

With the project plan in hand, the execution phase, involving simulation-based learning, ensues. Vigilant progress tracking against predefined success criteria is paramount, allowing real-time adjustments and effective issue mitigation. Transparent communication ensures alignment with scientific objectives. The focus is on systematic milestone achievement and deliverable generation, such as clinical knowledge acquisition (clinical objective) and enhanced student and faculty satisfaction (systematic objective). Regular progress meetings are instituted for stakeholder engagement, objective progress assessment, and expedited issue resolution.

##### Recommended tools

Project Management Software (e.g. Microsoft Project©), Communication Platforms (e.g. Microsoft Teams©).

This phase encompasses the core of our project—the execution of eight simulation-based training events. The subsequent section provides a concise yet detailed plan of the simulation-based training sessions.

### Clinical simulation training structure

We conduct a 2-hour simulation-based training session monthly featuring two high-fidelity simulation cases and debriefing with expert faculty. Out of the eight sessions, each addresses emergency situations prevalent in hospitals, with topics changing monthly. Each simulation scenario, lasting 20 min, accommodates up to four residents inside the simulation suits, while an average of 39 residents participates from an auditorium. During the simulation scenario, at least two tutors manage the case from the control room, and one tutor is present in the simulation room to adjust the scenario in case of difficulties. The requirement for each scenario is the occurrence of an actual acute emergency event. Examples include fainting patients after a hip replacement, septic patients, and patients with high fever or seizures. Each simulation case has a high degree of fidelity, replicating the clinical routine as realistically as possible. Standardization is maintained through a Simulation Case Charter detailing objectives, medical history, diagnostic and therapeutic pathways, and investigation printouts. Quality control is ensured by tutors familiar with case transcripts and checklists, supported by moderators, adept in leading debriefing sessions. A train-the-trainer course prepped tutors for comprehensive knowledge and quality control.

A pre-briefing is conducted before every session to ensure all tutors are familiar with the transcripts of the cases and the checklists. In addition, a train-the-trainer course is offered beforehand to all tutors to ensure adequate knowledge of the processes and to guarantee quality control. Experienced simulation tutors conduct the scenarios, and clinical consultants from the respective fields lead the debriefing discussions using relevant facts and guidelines.

### Performance evaluation

To evaluate the course’s effectiveness, we prepare a multiple-answer questionnaire consisting of ten questions each. The questions are designed in collaboration with the directors of the medical schools to assess knowledge acquisition and checked by three independent clinicians. The test is sent out electronically to all participants three hours before and immediately after the event. In parallel, all participants complete a self-report questionnaire evaluating clinical knowledge and nontechnical skills such as leadership, situational awareness, teamwork, and communication. The questionnaires are based on Kirkpatrick’s model for evaluating reactions and learning [22]. Satisfaction is evaluated based on a Likert scale. For the organizational evaluation, we conduct regular quality control meetings, review and document perceived risks and barriers during the implementation, and derive lessons learned. Team members can make suggestions for improvements based on their expert opinion and highlight risk areas to include in the risk logbook. The key recommendations and areas for improvement identified in these data are used to structure the lessons learned and risk mitigation reports. To capture the user’s perspective, we also conducted qualitative feedback surveys for faculty members, asking about their satisfaction with and evaluation of the implementation process.

### Data analysis

All the data collected by the faculty members and project manager are compiled for statistical analysis. The raw data are exported into Microsoft Excel® software (Microsoft Corporation, Redmond, WA, USA) and subsequently used to calculate descriptive statistics.

#### Phase 4: Closure phase

This stage is dedicated to the systematic resolution of all outstanding components. For formal acceptance, the conclusive deliverables are meticulously finalized and presented to stakeholders, including directors of residency schools and the university’s education manager. A comprehensive evaluation of the project’s achievements and setbacks is conducted, culminating in documenting valuable insights for future initiatives. This phase ensures the formal closure of the project, encompassing the presentation of outcomes to core stakeholders and team members.

##### Recommended tools

Project Review Template, Lessons Learned Document.

## Results

Adopting this structured four-phase approach, the subsequent section offers a descriptive analysis of the implementation pathway. Subsequently, quantitative feedback on the tangible impact of the simulation course on student performance is provided.

### Phase 1: Planning

Objectives: The *clinical objective* was to equip residents with knowledge on how to deal with common emergencies and increase their confidence in managing unpredictable events in different settings and with diverse teams. We decided to apply a pre-and post-test questionnaire to test the impact of the intervention on students’ confidence and skill acquisition. The results are displayed below. The *structural objective* was defined to address the effectiveness of the implementation process and comprises a qualitative evaluation. In the initial stages, we further engaged diverse stakeholders, comprising directors from selected residency schools (emergency medicine, anaesthesiology, surgery, gynaecology), one participant from communication and marketing, a scenario design team (instructional designer, several subject matter experts), and adjunct faculty, each with defined roles and responsibilities (refer to the appendix). The project manager played a crucial role in overseeing the planning, execution, and successful completion of the project. Notably, external stakeholders, such as medical societies and potential course sponsors, were separate from this project but should be considered for future projects.

### Phase 2: Build-up

During the build-up phase, the strategic direction was translated into a schedule of eight simulation sessions, and we developed a precise curriculum, implementing it in the third stage. A project monitoring log was established, delineating processes for communication, marketing, enrolment handling, simulation scenarios, and budgeting. Weekly core team meetings, comprising the design team member, a technician, and two tutors, addressed progress, barriers, timelines, potential risks, and milestones, documented in a risk mitigation logbook.

Challenges in this phase were predominantly associated with marketing and communication, necessitating the identification of efficient awareness-building channels and increasing director buy-in. The initial email campaign expanded to include WhatsApp (WhatsApp LLC, Menlo Park, CA, USA) messaging, social media, and presence at monthly morbidity and mortality meetings. We further used billboards and a traditional newsletter to optimize general program awareness. Despite starting with an inclusive approach to involve all residents at Humanitas University, we observed a decline in participation due to shifting priorities towards increased clinical responsibilities. Consequently, we shifted our communication strategy to a “top-down” approach, incentivizing residency school directors, significantly enhancing buy-in, and achieving sustainable participation rates. Key incentives included integrating simulation courses into the national curriculum, allowing students to document activities in their national logbook, and freeing training time for clinical learning in the hospital. Furthermore, we actively involved the directors in the scientific research program, showcasing real-time data analytics outputs and the ongoing performance improvements of the residents. This engagement gave the directors firsthand insights into the program’s outcomes and the residents’ progress.

### Phase 3: Execution phase

Mid-2021 we transitioned into the execution phase, where the initial eight simulation-based training sessions were conducted. Each two-hour session followed the structure of running two simulated clinical cases for 20 min, followed by a 60-minute expert-led debriefing session in the auditorium. Further details on these sessions can be found in the preceding chapter. We conducted knowledge assessments, and within the first 12 months, we transitioned from an email reminder to a live evaluation format using Wooclap® Online Quiz. This modification in the evaluation mode aimed to enhance the assessment process, adapt to a more interactive and dynamic approach, and ultimately lead to a lower attrition rate.

### Phase 4: Closing

In the final closing phase, aimed at closing and reviewing the achievements of key objectives, we conducted a comprehensive review of the performance and outcomes. We identified areas of improvement (see below) and finalized the lessons learned to refine the potential scalability of the project:

## Educational evaluation and outcomes

Three hundred ninety-five residents participated in the monthly simulation-based events, with an average participation rate of 39 (+/- 19 residents) per lecture. The average rating for expected skills learned significantly improved from 5.9/10 to 8.8/10 (*p* = 0.0001). Participants found the impact on future clinical practice (4,7/5) and teamwork (4.8/5) most helpful. Faculty members appreciated the new organizational structure, giving them more visibility and time savings (4,8/5). 98% of the residents expressed interest in having more high-fidelity simulation teaching during their residency programs.

## Implementation evaluation and outcomes

In the final closing phase, we developed a post-evaluation report focusing on the insights gained throughout the phases in order to address scepticism, increase program awareness, and optimize operational performance.

### Focus 1: Awareness and skepticism

#### Residents and directors buy-in

In the initial phase, we started to target our marketing campaign directly to residents. We changed course and initiated a top-down approach, actively involving the Leadership (directors and representatives) of the respective schools. Support and direct communication with residents’ representatives and directors were most efficient at ensuring high participation rates. We involved the directors in our research studies and regularly presented our scientifically founded data on training effectiveness.

#### Aligning policies and procedures

To fulfil residency requirements, each resident must undertake a specified number of training activities, all meticulously documented in a national electronic registry known as the “libretto.” To bolster participation, we implemented successful modifications to organizational policies, including SIMCLUB simulation activities within the electronic register. This pivotal stage demanded a meticulous understanding and adherence to accrediting standards and regulatory requirements.

#### Building awareness and reputation

We started our internal communication strategy with email information about course enrolment. Early on, we decided to add a more progressive approach, including communication such as social media marketing, WhatsApp groups, and billboard visualization. The communication department’s involvement was key to devolving the messages and benefits of the training program.

#### Understanding motivators and organizational culture

It was essential to understand the motivators of critical stakeholders and their alignment with the organization’s values, beliefs, and behaviours, and the organizational DNA. We continuously communicated the benefits of simulation for patient care and the impact on the university’s reputation as a leader in innovative training concepts.

### Operational performance

#### Introducing interactivity and gamification

We quickly realized we had a high attrition rate for residents’ answers to our “pre- and post-test” evaluation forms. Hence, after the initial trial period, we converted our evaluation procedure to a “live voting” system. This was performed during the simulation session based on a Q.R. code-accessible online quiz. In addition, after each session, we awarded the leaderboard winner a prize (usually a university gadget). These actions decreased our attrition rate from 39–9%. Moreover, we focused on personalizing debriefing and inviting an expert table and directors from various specialties to allow for a profound educational experience.

#### Cooperatively defining and early communicating the project scope

It was important to focus on the most important objectives that align with the educational goals of the simulation-based curriculum, and design the project plan accordingly. By communicating and prioritizing educational priorities, Faculty members can allocate their time and effort more efficiently.

#### Creating and adhering to the project plan

Early on, we created an elaborate plan delineating tasks, timelines, and requisite resources for completion. We used Project Management Software, such as Microsoft Project©, to monitor progress and identify overlooked milestones. Examples encompass a meticulous scheduling plan to optimize faculty time during simulations, ensuring efficient time management. This facilitated the adherence to timelines, provided alerts for overdue tasks, and communicated delays promptly to teams or individuals.

#### Costs

The main challenges were related to running the simulation. We relied on the in-kind contributions of faculty, who dedicated time and effort to their free time. We created a library of simulation cases, trailed blended learning concepts, and cooperated with other institutions to bundle resources. However, the cost-effectiveness of these methods needs to be evaluated. External funding, such as grants or partnerships, may assist with future projects.

### Risks and mitigation strategies

Risks were identified during all phases. Each team member had access to the risk log, to independently log perceived risks, which were later discussed and grouped into three groups. This was followed by the development of mitigation strategies important for preventing pitfalls. Table 1 summarises the most prominent risks in implementing such an educational program and the mitigation strategies we applied.

**Table 1:** Perceived risks and mitigation strategies

Encountered risks	Mitigation strategy
**Technical Risks**	
Compatibility issues: The simulation software may be compatible with the existing technology infrastructure, such as hardware, operating systems, or network configurations.	Conducting Pilots: We scheduled a dry run before every simulation with our engineer to check technical issues.
System failures: There is a risk of technical glitches, software bugs, or system crashes that could disrupt the training sessions and negatively impact the learning experience.	
Data security: Storing and handling sensitive participant data within the simulation system may pose risks if proper data security measures are not in place.	Collaborate with the I.T. department to address data security risks and find secure storage space within our internal platform.
**Resource Risks**	
Lack of skilled personnel: Difficulty finding and training trainers or facilitators proficient in using the simulation software and delivering practical training sessions.	Providing adequate training and support: We installed a Train the Trainer course, reserved for future simulation consultants, and adjusted to the specific needs of the SIMCLUB (e.g., the transversal character of the cases)
Time constraints: Inadequate time for course development, testing, and customization of the simulation-based training materials and scenarios.	We partnered with the course directors to have a dedicated simulation champion in each department, which had reserved time and expertise in and for simulation.
**User Adoption Risks**	
Resistance to change: Participants or trainers may resist adopting the new simulation-based approach due to unfamiliarity, scepticism, or preference for traditional training methods.	After each session, we presented data on outcomes (satisfaction and learning effect). This was visualized together with the international literature on simulation-based training effects and patient outcome improvement.
Technical proficiency: Participants may face challenges adjusting to a simulation scenario, hindering their engagement, and learning outcomes.	We tried to replicate the hospital setting as realistic as possible. Before every scenario, we briefed the participants to assume a real case, to speak, touch, and carefully listen to the “patient.” Moreover, we introduced actors to create stressful and realistic scenarios.

## Discussion

Applying the Harvard HBR Project Management Approach in developing a simulation-based medical curriculum at Humanitas University yielded valuable insights into enhancing medical education. The integration of project management strategies, rooted in a structured approach, facilitated the successful implementation of a training curriculum, significantly enhancing residents’ skills. Evidenced by a substantial increase in average knowledge gain from 5.9/10 to 8.8/10, these results affirm the program’s efficacy. However, exploring implementation science in medical simulation remains neglected in the existing literature. Our study embraced a systematic approach, leveraging the Harvard Project Management Theory to identify foundational pillars and critical elements within implementation science and traced the impact on students’ performance. This structured method encompassed the evaluation of faculty time, incentives, motivation, resources, and organizational policies.

Previous research has emphasized the critical role of systematic frameworks and dedicated implementation teams in positively influencing educational outcomes [23–25]. In a review of 59 studies, DuBois et al. [26] reported a significant relationship between the monitoring implementation process and the final effect size (mean effect of 0.18 vs. 0.06). Durlak et al. confirmed these findings, offering strong evidence that programs implemented by well-defined implementation teams take approximately three years to fully implement, with an 80% success rate. In comparison, programs without such teams take approximately 17 years, with a success rate of only 14% [25]. Those results underscored the correlation between the implementation process and final effect size, emphasizing the significance of well-defined teams and structures for successful program implementation.

Notably, our study highlighted stakeholder engagement, change management, and ongoing data analytics and presentation as pivotal components contributing to the success of our project. These elements were vital in mitigating perceived barriers such as scepticism, buy-in, or time constraints. Stakeholder engagement emerged as the most demanding and rewarding aspect of the implementation process. Previous studies have demonstrated the positive influence of student engagement on the ultimate learning outcomes, particularly in the context of clinical practice, underscoring the significance of effective engagement strategies [27]. Additionally, inspired by established practices in the business domain [28], our emphasis extended to comprehending motivators and organizational culture. This included ensuring alignment with stakeholders’ values, accentuating the benefits of simulation for patient care, and enhancing the university’s standing in pioneering training concepts.

Successfully disseminating awareness through diverse communication channels and aligning individual motivators with organizational values proved effective in garnering buy-in and support. In alignment with recent literature emphasizing the importance of addressing resistance to change through a framework [29], with communication being a pivotal factor, our strategic emphasis extended to raising awareness through progressive communication channels such as email, social media, WhatsApp groups, and billboards. Furthermore, incentivizing stakeholders by advocating for the incorporation of simulation activities in the national electronic registry, aligning policies, and encouraging resident participation notably enhanced overall participation rates.

Finally, the introduction of interactivity and gamification techniques, such as live voting and the provision of prizes, played a crucial role in increasing participant involvement and reducing attrition rates. While recent studies have debated the sustainability of gamification on educational outcomes [30, 31], we observed an apparent increase in test participation and a decrease in the attrition rate from 39 to 9%.

Despite these positive outcomes, the study has limitations, including its single-institution focus. Replicating the study in diverse healthcare environments would provide a more comprehensive understanding of the effectiveness and challenges of implementing similar programs. Additionally, a thorough cost-effectiveness analysis was not conducted, and caution is advised when interpreting potential costs and resource utilization.

## Conclusion

In conclusion, implementing the Harvard Project Management Approach in designing and implementing a simulation-based medical curriculum offers valuable lessons and practical recommendations for enhancing curriculum design success and improving emergency preparedness among medical residents. The positive outcomes observed in this study highlight the importance of adopting a structured and systematic approach to curriculum design, with project management strategies playing a crucial role in optimizing efficiency, acceptability, and sustainability. By incorporating project management tools and principles, medical education programs can effectively address the challenges of implementing simulation-based training and provide residents with an immersive and impactful learning experience.

### Electronic supplementary material

Below is the link to the electronic supplementary material.

Supplementary Material 1

## Data Availability

The datasets used and analysed during the current study are available from the corresponding author upon reasonable request.
