# Supplementary material for: Unleashing excellence: using a project management approach to effectively implement a simulation curriculum to improve residents’ preparedness
Source: BMC Med Educ. 2024 Mar 4;24:234. doi: 10.1186/s12909-024-05166-y (PMC10913544; doi:10.1186/s12909-024-05166-y)
Supplement: Supplementary file 1 — Supplementary Material 1 [file 12909_2024_5166_MOESM1_ESM.doc]

**STAKEHOLDER MAP**

Appendix: this figure shows the stakeholders involved I the project, and their respective roles.

| **Stakeholder** | **Role** |
| --- | --- |
| Marketing and Communication | Oversees the entire curriculum design process and ensures that the project stays on schedule and within budget. |
| Simulation Designer | Designs the overall structure and content of the curriculum. Overseas the flow of the actual simulation scenarios. |
| Sim Tutors | Runs the actual simulations. Prepares, together with the Designers, the scenario, and interfaces with the medical directors to adhere to scientific standards. |
| Content Experts | Depending on the simulation topic, we asked clinicians to contribute their knowledge |
| Directors | Proposing the educational direction, and delegating educational tasks to team clinicians |
| Manager of Educational Learning | Responsible for the residency schools educational learning. Supports the buy-in and build awareness for the university’s innovative offerings |
| CEO | Confirms the project. |
| IT / Technicians | Support the infrastructure which enables the sim scenarios to run. |
